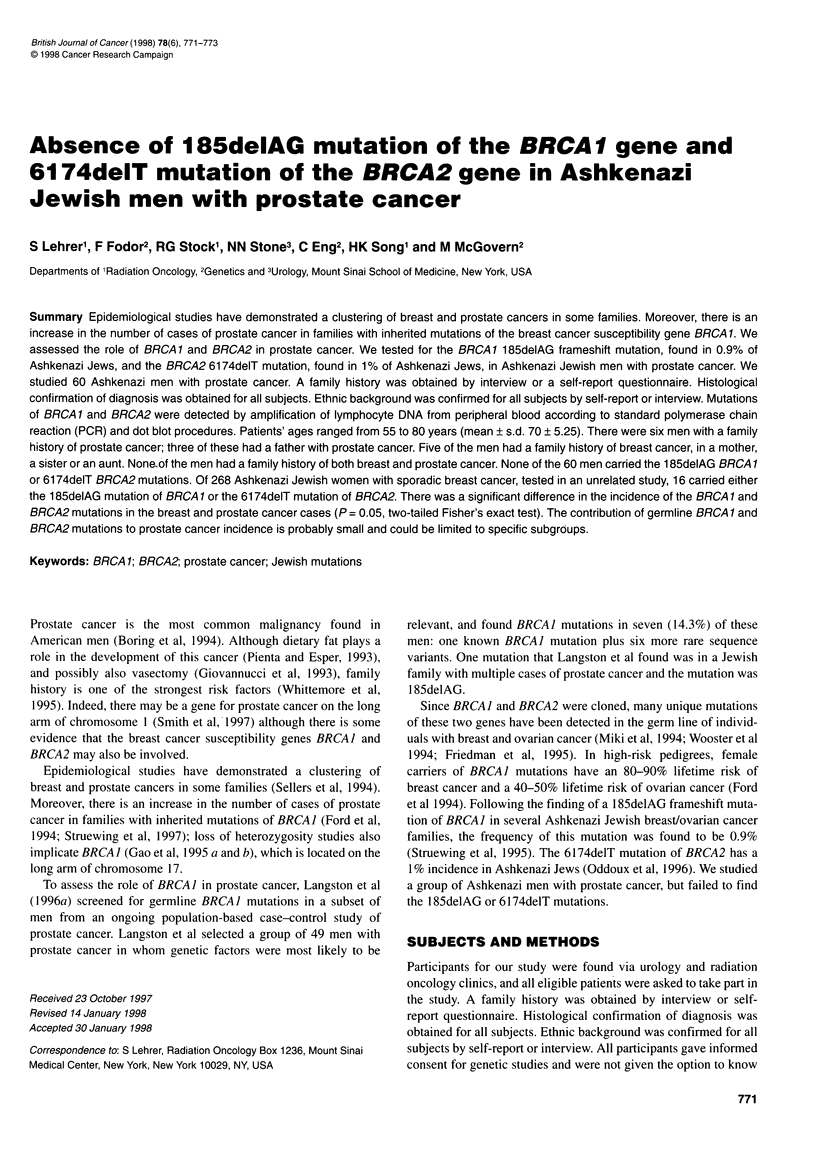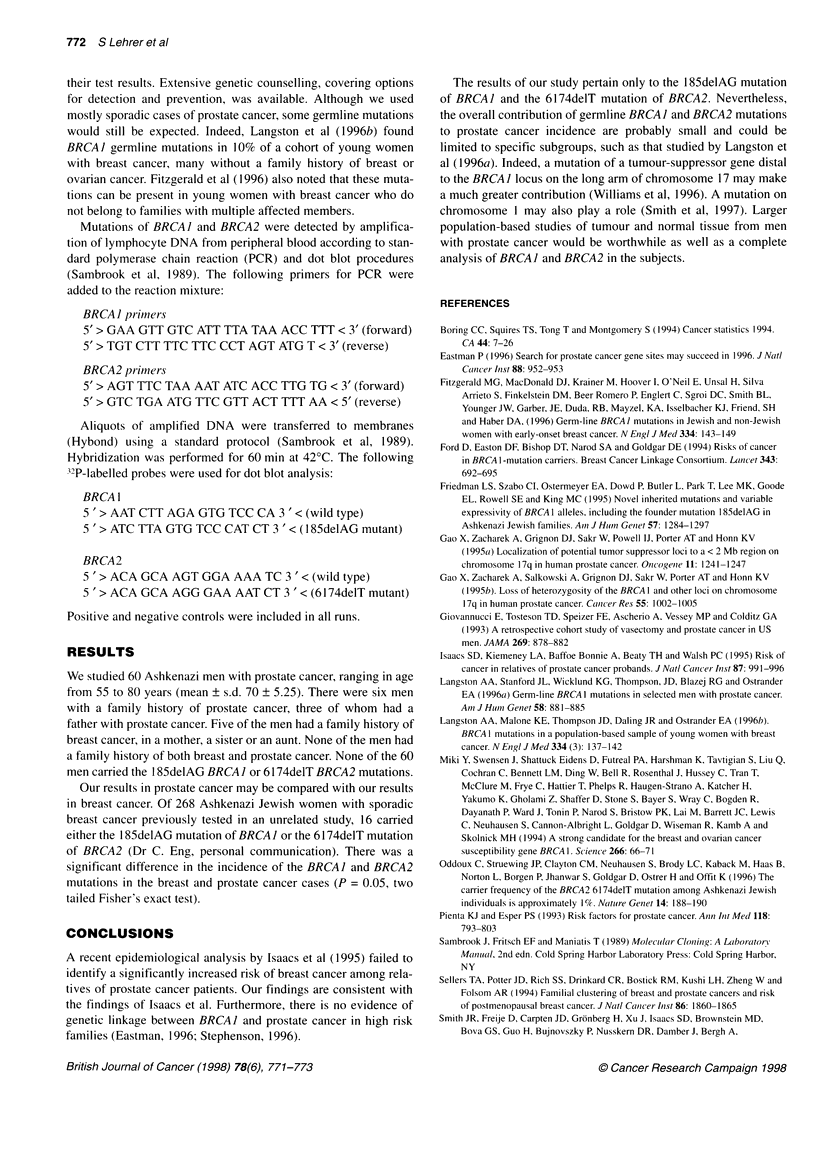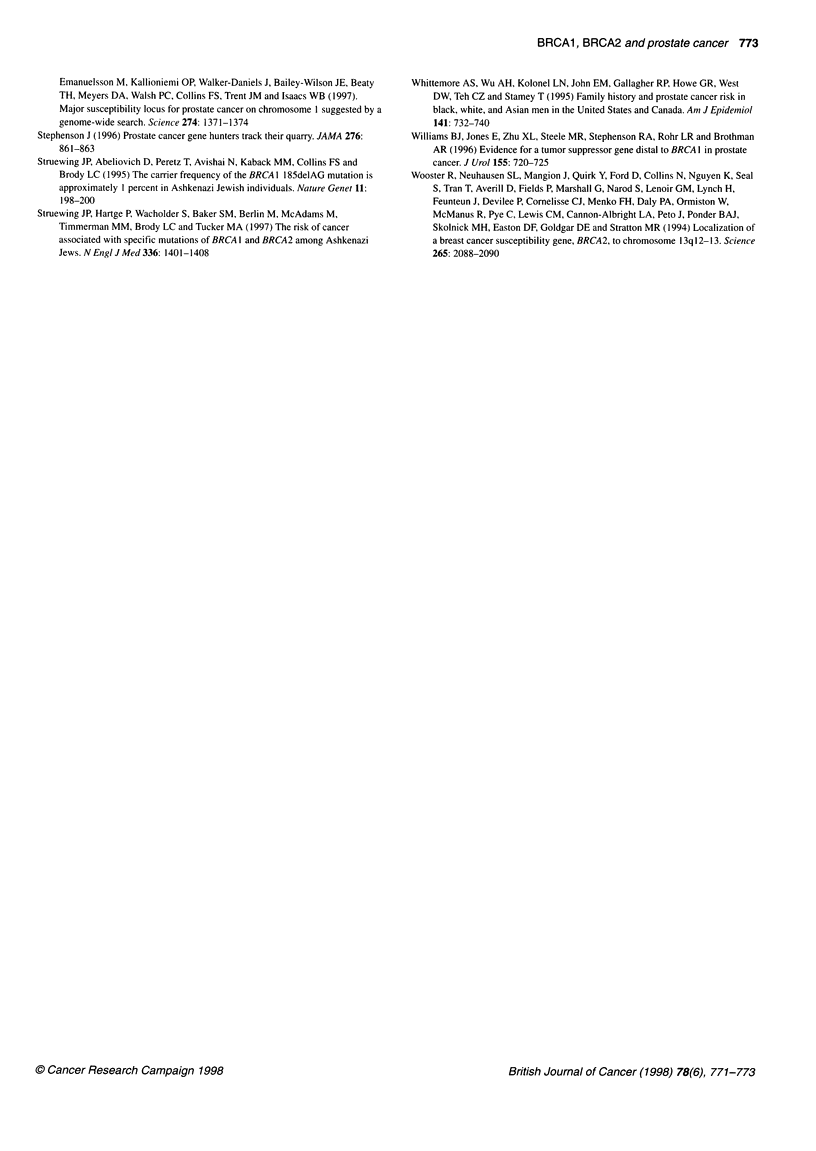# Absence of 185delAG mutation of the BRCA1 gene and 6174delT mutation of the BRCA2 gene in Ashkenazi Jewish men with prostate cancer.

**DOI:** 10.1038/bjc.1998.576

**Published:** 1998-09

**Authors:** S. Lehrer, F. Fodor, R. G. Stock, N. N. Stone, C. Eng, H. K. Song, M. McGovern

**Affiliations:** Department of Radiation Oncology, Mount Sinai School of Medicine, New York, New York 10029, USA.

## Abstract

Epidemiological studies have demonstrated a clustering of breast and prostate cancers in some families. Moreover, there is an increase in the number of cases of prostate cancer in families with inherited mutations of the breast cancer susceptibility gene BRCA1. We assessed the role of BRCA1 and BRCA2 in prostate cancer. We tested for the BRCA1 185delAG frameshift mutation, found in 0.9% of Ashkenazi Jews, and the BRCA2 6174delT mutation, found in 1% of Ashkenazi Jews, in Ashkenazi Jewish men with prostate cancer. We studied 60 Ashkenazi men with prostate cancer. A family history was obtained by interview or a self-report questionnaire. Histological confirmation of diagnosis was obtained for all subjects. Ethnic background was confirmed for all subjects by self-report or interview. Mutations of BRCA1 and BRCA2 were detected by amplification of lymphocyte DNA from peripheral blood according to standard polymerase chain reaction (PCR) and dot blot procedures. Patients' ages ranged from 55 to 80 years (mean +/- s.d. 70 +/- 5.25). There were six men with a family history of prostate cancer; three of these had a father with prostate cancer. Five of the men had a family history of breast cancer, in a mother, a sister or an aunt. None of the men had a family history of both breast and prostate cancer. None of the 60 men carried the 185delAG BRCA1 or 6174delT BRCA2 mutations. Of 268 Ashkenazi Jewish women with sporadic breast cancer, tested in an unrelated study, 16 carried either the 185delAG mutation of BRCA1 or the 6174delT mutation of BRCA2. There was a significant difference in the incidence of the BRCA1 and BRCA2 mutations in the breast and prostate cancer cases (P = 0.05, two-tailed Fisher's exact test). The contribution of germline BRCA1 and BRCA2 mutations to prostate cancer incidence is probably small and could be limited to specific subgroups.


British Journal of Cancer (1998) 78(6), 771–773
© 1998 Cancer Research Campaign

### Absence of 185delAG mutation of the *BRCA1* gene and 6174delT mutation of the *BRCA2* gene in Ashkenazi Jewish men with prostate cancer

**S Lehrer[1], F Fodor[2], RG Stock[1], NN Stone[3], C Eng[2], HK Song[1] and M McGovern[2]**

Departments of [1]Radiation Oncology, [2]Genetics and [3]Urology, Mount Sinai School of Medicine, New York, USA

**Summary** Epidemiological studies have demonstrated a clustering of breast and prostate cancers in some families. Moreover, there is an increase in the number of cases of prostate cancer in families with inherited mutations of the breast cancer susceptibility gene *BRCA1*. We assessed the role of *BRCA1* and *BRCA2* in prostate cancer. We tested for the *BRCA1* 185delAG frameshift mutation, found in 0.9% of Ashkenazi Jews, and the *BRCA2* 6174delT mutation, found in 1% of Ashkenazi Jews, in Ashkenazi Jewish men with prostate cancer. We studied 60 Ashkenazi men with prostate cancer. A family history was obtained by interview or a self-report questionnaire. Histological confirmation of diagnosis was obtained for all subjects. Ethnic background was confirmed for all subjects by self-report or interview. Mutations of *BRCA1* and *BRCA2* were detected by amplification of lymphocyte DNA from peripheral blood according to standard polymerase chain reaction (PCR) and dot blot procedures. Patients' ages ranged from 55 to 80 years (mean ± s.d. 70 ± 5.25). There were six men with a family history of prostate cancer; three of these had a father with prostate cancer. Five of the men had a family history of breast cancer, in a mother, a sister or an aunt. None of the men had a family history of both breast and prostate cancer. None of the 60 men carried the 185delAG *BRCA1* or 6174delT *BRCA2* mutations. Of 268 Ashkenazi Jewish women with sporadic breast cancer, tested in an unrelated study, 16 carried either the 185delAG mutation of *BRCA1* or the 6174delT mutation of *BRCA2*. There was a significant difference in the incidence of the *BRCA1* and *BRCA2* mutations in the breast and prostate cancer cases ($P = 0.05$, two-tailed Fisher's exact test). The contribution of germline *BRCA1* and *BRCA2* mutations to prostate cancer incidence is probably small and could be limited to specific subgroups.

**Keywords:** *BRCA1*; *BRCA2*; prostate cancer; Jewish mutations

Prostate cancer is the most common malignancy found in American men (Boring et al, 1994). Although dietary fat plays a role in the development of this cancer (Pienta and Esper, 1993), and possibly also vasectomy (Giovannucci et al, 1993), family history is one of the strongest risk factors (Whittemore et al, 1995). Indeed, there may be a gene for prostate cancer on the long arm of chromosome 1 (Smith et al, 1997) although there is some evidence that the breast cancer susceptibility genes *BRCA1* and *BRCA2* may also be involved.

Epidemiological studies have demonstrated a clustering of breast and prostate cancers in some families (Sellers et al, 1994). Moreover, there is an increase in the number of cases of prostate cancer in families with inherited mutations of *BRCA1* (Ford et al, 1994; Struewing et al, 1997); loss of heterozygosity studies also implicate *BRCA1* (Gao et al, 1995 *a* and *b*), which is located on the long arm of chromosome 17.

To assess the role of *BRCA1* in prostate cancer, Langston et al (1996*a*) screened for germline *BRCA1* mutations in a subset of men from an ongoing population-based case–control study of prostate cancer. Langston et al selected a group of 49 men with prostate cancer in whom genetic factors were most likely to be relevant, and found *BRCA1* mutations in seven (14.3%) of these men: one known *BRCA1* mutation plus six more rare sequence variants. One mutation that Langston et al found was in a Jewish family with multiple cases of prostate cancer and the mutation was 185delAG.

Since *BRCA1* and *BRCA2* were cloned, many unique mutations of these two genes have been detected in the germ line of individuals with breast and ovarian cancer (Miki et al, 1994; Wooster et al 1994; Friedman et al, 1995). In high-risk pedigrees, female carriers of *BRCA1* mutations have an 80–90% lifetime risk of breast cancer and a 40–50% lifetime risk of ovarian cancer (Ford et al 1994). Following the finding of a 185delAG frameshift mutation of *BRCA1* in several Ashkenazi Jewish breast/ovarian cancer families, the frequency of this mutation was found to be 0.9% (Struewing et al, 1995). The 6174delT mutation of *BRCA2* has a 1% incidence in Ashkenazi Jews (Oddoux et al, 1996). We studied a group of Ashkenazi men with prostate cancer, but failed to find the 185delAG or 6174delT mutations.

### SUBJECTS AND METHODS

Participants for our study were found via urology and radiation oncology clinics, and all eligible patients were asked to take part in the study. A family history was obtained by interview or self-report questionnaire. Histological confirmation of diagnosis was obtained for all subjects. Ethnic background was confirmed for all subjects by self-report or interview. All participants gave informed consent for genetic studies and were not given the option to know

*Received 23 October 1997*
*Revised 14 January 1998*
*Accepted 30 January 1998*

*Correspondence to*: S Lehrer, Radiation Oncology Box 1236, Mount Sinai Medical Center, New York, New York 10029, NY, USA

their test results. Extensive genetic counselling, covering options for detection and prevention, was available. Although we used mostly sporadic cases of prostate cancer, some germline mutations would still be expected. Indeed, Langston et al (1996*b*) found *BRCA1* germline mutations in 10% of a cohort of young women with breast cancer, many without a family history of breast or ovarian cancer. Fitzgerald et al (1996) also noted that these mutations can be present in young women with breast cancer who do not belong to families with multiple affected members.

Mutations of *BRCA1* and *BRCA2* were detected by amplification of lymphocyte DNA from peripheral blood according to standard polymerase chain reaction (PCR) and dot blot procedures (Sambrook et al, 1989). The following primers for PCR were added to the reaction mixture:

*BRCA1 primers*
5′ > GAA GTT GTC ATT TTA TAA ACC TTT < 3′ (forward)
5′ > TGT CTT TTC TTC CCT AGT ATG T < 3′ (reverse)

*BRCA2 primers*
5′ > AGT TTC TAA AAT ATC ACC TTG TG < 3′ (forward)
5′ > GTC TGA ATG TTC GTT ACT TTT AA < 5′ (reverse)

Aliquots of amplified DNA were transferred to membranes (Hybond) using a standard protocol (Sambrook et al, 1989). Hybridization was performed for 60 min at 42°C. The following $^{32}$P-labelled probes were used for dot blot analysis:

*BRCA*1
5 ′ > AAT CTT AGA GTG TCC CA 3 ′ < (wild type)
5 ′ > ATC TTA GTG TCC CAT CT 3 ′ < (185delAG mutant)

*BRCA*2
5 ′ > ACA GCA AGT GGA AAA TC 3 ′ < (wild type)
5 ′ > ACA GCA AGG GAA AAT CT 3 ′ < (6174delT mutant)

Positive and negative controls were included in all runs.

### RESULTS

We studied 60 Ashkenazi men with prostate cancer, ranging in age from 55 to 80 years (mean ± s.d. 70 ± 5.25). There were six men with a family history of prostate cancer, three of whom had a father with prostate cancer. Five of the men had a family history of breast cancer, in a mother, a sister or an aunt. None of the men had a family history of both breast and prostate cancer. None of the 60 men carried the 185delAG *BRCA1* or 6174delT *BRCA2* mutations.

Our results in prostate cancer may be compared with our results in breast cancer. Of 268 Ashkenazi Jewish women with sporadic breast cancer previously tested in an unrelated study, 16 carried either the 185delAG mutation of *BRCA1* or the 6174delT mutation of *BRCA2* (Dr C. Eng, personal communication). There was a significant difference in the incidence of the *BRCA1* and *BRCA2* mutations in the breast and prostate cancer cases ($P = 0.05$, two tailed Fisher's exact test).

### CONCLUSIONS

A recent epidemiological analysis by Isaacs et al (1995) failed to identify a significantly increased risk of breast cancer among relatives of prostate cancer patients. Our findings are consistent with the findings of Isaacs et al. Furthermore, there is no evidence of genetic linkage between *BRCA1* and prostate cancer in high risk families (Eastman, 1996; Stephenson, 1996).

The results of our study pertain only to the 185delAG mutation of *BRCA1* and the 6174delT mutation of *BRCA2*. Nevertheless, the overall contribution of germline *BRCA1* and *BRCA2* mutations to prostate cancer incidence are probably small and could be limited to specific subgroups, such as that studied by Langston et al (1996*a*). Indeed, a mutation of a tumour-suppressor gene distal to the *BRCA1* locus on the long arm of chromosome 17 may make a much greater contribution (Williams et al, 1996). A mutation on chromosome 1 may also play a role (Smith et al, 1997). Larger population-based studies of tumour and normal tissue from men with prostate cancer would be worthwhile as well as a complete analysis of *BRCA1* and *BRCA2* in the subjects.

### REFERENCES

Boring CC, Squires TS, Tong T and Montgomery S (1994) Cancer statistics 1994. *CA* **44**: 7–26

Eastman P (1996) Search for prostate cancer gene sites may succeed in 1996. *J Natl Cancer Inst* **88**: 952–953

Fitzgerald MG, MacDonald DJ, Krainer M, Hoover I, O'Neil E, Unsal H, Silva Arrieto S, Finkelstein DM, Beer Romero P, Englert C, Sgroi DC, Smith BL, Younger JW, Garber, JE, Duda, RB, Mayzel, KA, Isselbacher KJ, Friend, SH and Haber DA, (1996) Germ-line *BRCA1* mutations in Jewish and non-Jewish women with early-onset breast cancer. *N Engl J Med* **334**: 143–149

Ford D, Easton DF, Bishop DT, Narod SA and Goldgar DE (1994) Risks of cancer in *BRCA*1-mutation carriers. Breast Cancer Linkage Consortium. *Lancet* **343**: 692–695

Friedman LS, Szabo CI, Ostermeyer EA, Dowd P, Butler L, Park T, Lee MK, Goode EL, Rowell SE and King MC (1995) Novel inherited mutations and variable expressivity of *BRCA*1 alleles, including the founder mutation 185delAG in Ashkenazi Jewish families. *Am J Hum Genet* **57**: 1284–1297

Gao X, Zacharek A, Grignon DJ, Sakr W, Powell IJ, Porter AT and Honn KV (1995*a*) Localization of potential tumor suppressor loci to a < 2 Mb region on chromosome 17q in human prostate cancer. *Oncogene* **11**: 1241–1247

Gao X, Zacharek A, Salkowski A, Grignon DJ, Sakr W, Porter AT and Honn KV (1995*b*). Loss of heterozygosity of the *BRCA*1 and other loci on chromosome 17q in human prostate cancer. *Cancer Res* **55**: 1002–1005

Giovannucci E, Tosteson TD, Speizer FE, Ascherio A, Vessey MP and Colditz GA (1993) A retrospective cohort study of vasectomy and prostate cancer in US men. *JAMA* **269**: 878–882

Isaacs SD, Kiemeney LA, Baffoe Bonnie A, Beaty TH and Walsh PC (1995) Risk of cancer in relatives of prostate cancer probands. *J Natl Cancer Inst* **87**: 991–996

Langston AA, Stanford JL, Wicklund KG, Thompson, JD, Blazej RG and Ostrander EA (1996*a*) Germ-line *BRCA*1 mutations in selected men with prostate cancer. *Am J Hum Genet* **58**: 881–885

Langston AA, Malone KE, Thompson JD, Daling JR and Ostrander EA (1996*b*). *BRCA*1 mutations in a population-based sample of young women with breast cancer. *N Engl J Med* **334** (3): 137–142

Miki Y, Swensen J, Shattuck Eidens D, Futreal PA, Harshman K, Tavtigian S, Liu Q, Cochran C, Bennett LM, Ding W, Bell R, Rosenthal J, Hussey C, Tran T, McClure M, Frye C, Hattier T, Phelps R, Haugen-Strano A, Katcher H, Yakumo K, Gholami Z, Shaffer D, Stone S, Bayer S, Wray C, Bogden R, Dayanath P, Ward J, Tonin P, Narod S, Bristow PK, Lai M, Barrett JC, Lewis C, Neuhausen S, Cannon-Albright L, Goldgar D, Wiseman R, Kamb A and Skolnick MH (1994) A strong candidate for the breast and ovarian cancer susceptibility gene *BRCA*1. *Science* **266**: 66–71

Oddoux C, Struewing JP, Clayton CM, Neuhausen S, Brody LC, Kaback M, Haas B, Norton L, Borgen P, Jhanwar S, Goldgar D, Ostrer H and Offit K (1996) The carrier frequency of the *BRCA*2 6174delT mutation among Ashkenazi Jewish individuals is approximately 1%. *Nature Genet* **14**: 188–190

Pienta KJ and Esper PS (1993) Risk factors for prostate cancer. *Ann Int Med* **118**: 793–803

Sambrook J, Fritsch EF and Maniatis T (1989) *Molecular Cloning: A Laboratory Manual*, 2nd edn. Cold Spring Harbor Laboratory Press: Cold Spring Harbor, NY

Sellers TA, Potter JD, Rich SS, Drinkard CR, Bostick RM, Kushi LH, Zheng W and Folsom AR (1994) Familial clustering of breast and prostate cancers and risk of postmenopausal breast cancer. *J Natl Cancer Inst* **86**: 1860–1865

Smith JR, Freije D, Carpten JD, Grönberg H, Xu J, Isaacs SD, Brownstein MD, Bova GS, Guo H, Bujnovszky P, Nusskern DR, Damber J, Bergh A,

*© Cancer Research Campaign 1998*

Emanuelsson M, Kallioniemi OP, Walker-Daniels J, Bailey-Wilson JE, Beaty TH, Meyers DA, Walsh PC, Collins FS, Trent JM and Isaacs WB (1997). Major susceptibility locus for prostate cancer on chromosome 1 suggested by a genome-wide search. *Science* **274**: 1371–1374

Stephenson J (1996) Prostate cancer gene hunters track their quarry. *JAMA* **276**: 861–863

Struewing JP, Abeliovich D, Peretz T, Avishai N, Kaback MM, Collins FS and Brody LC (1995) The carrier frequency of the *BRCA*1 185delAG mutation is approximately 1 percent in Ashkenazi Jewish individuals. *Nature Genet* **11**: 198–200

Struewing JP, Hartge P, Wacholder S, Baker SM, Berlin M, McAdams M, Timmerman MM, Brody LC and Tucker MA (1997) The risk of cancer associated with specific mutations of *BRCA*1 and *BRCA*2 among Ashkenazi Jews. *N Engl J Med* **336**: 1401–1408

Whittemore AS, Wu AH, Kolonel LN, John EM, Gallagher RP, Howe GR, West DW, Teh CZ and Stamey T (1995) Family history and prostate cancer risk in black, white, and Asian men in the United States and Canada. *Am J Epidemiol* **141**: 732–740

Williams BJ, Jones E, Zhu XL, Steele MR, Stephenson RA, Rohr LR and Brothman AR (1996) Evidence for a tumor suppressor gene distal to *BRCA*1 in prostate cancer. *J Urol* **155**: 720–725

Wooster R, Neuhausen SL, Mangion J, Quirk Y, Ford D, Collins N, Nguyen K, Seal S, Tran T, Averill D, Fields P, Marshall G, Narod S, Lenoir GM, Lynch H, Feunteun J, Devilee P, Cornelisse CJ, Menko FH, Daly PA, Ormiston W, McManus R, Pye C, Lewis CM, Cannon-Albright LA, Peto J, Ponder BAJ, Skolnick MH, Easton DF, Goldgar DE and Stratton MR (1994) Localization of a breast cancer susceptibility gene, *BRCA*2, to chromosome 13q12-13. *Science* **265**: 2088–2090

© Cancer Research Campaign 1998